# Stabilization and Release of Palm Tocotrienol Emulsion Fabricated Using pH-Sensitive Calcium Carbonate

**DOI:** 10.3390/foods10020358

**Published:** 2021-02-07

**Authors:** Phui Yee Tan, Beng Ti Tey, Eng Seng Chan, Oi Ming Lai, Hon Weng Chang, Tai Boon Tan, Yuanfa Liu, Yong Wang, Chin Ping Tan

**Affiliations:** 1 Department of Bioscience, Faculty of Applied Sciences, Tunku Abdul Rahman University College, Jalan Genting Kelang, Setapak, Kuala Lumpur 53300, Malaysia; phuiyee@tarc.edu.my; 2 Chemical Engineering Discipline, School of Engineering, Monash University Malaysia, Jalan Lagoon Selatan, Bandar Sunway 47500, Selangor, Malaysia; tey.beng.ti@monash.edu (B.T.T.); chan.eng.seng@monash.edu (E.S.C.); 3 Monash-Industry Palm Oil Education and Research Platform (MIPO), Monash University Malaysia, Jalan Lagoon Selatan, Bandar Sunway 47500, Selangor, Malaysia; 4 Department of Bioprocess Technology, Faculty of Biotechnology and Biomolecular Sciences, Universiti Putra Malaysia, UPM, Serdang 43400, Selangor, Malaysia; omlai@upm.edu.my; 5 Department of Food Technology, Faculty of Food Science and Technology, Universiti Putra Malaysia, UPM, Serdang 43400, Selangor, Malaysia; honweng90@live.com; 6 Department of Food Service and Management, Faculty of Food Science and Technology, Universiti Putra Malaysia, Serdang 43400 UPM, Selangor, Malaysia; taiboon_tan@upm.edu.my; 7 State Key Laboratory of Food Science and Technology, School of Food Science and Technology, National Engineering Research Center for Functional Food, Jiangnan University, Wuxi 214122, China; yfliu@jiangnan.edu.cn; 8 Collaborative Innovation Center of Food Safety and Quality Control in Jiangsu Province, National Engineering Laboratory for Cereal Fermentation Technology, Jiangnan University, Wuxi 214122, China; 9 JNU-UPM International Joint Laboratory on Plant Oil Processing and Safety (POPS), Department of Food Science and Engineering, Jinan University, Guangzhou 510632, China; twyong@jnu.edu.cn; 10 Laboratory of Processing and Product Development, Institute of Plantation Studies, Universiti Putra Malaysia, UPM, Serdang 43400, Malaysia

**Keywords:** tocotrienols, pickering emulsion, self-assembly, stability, entrapment efficiency, homogenization

## Abstract

Calcium carbonate (CaCO_3_) has been utilized as a pH-responsive component in various products. In this present work, palm tocotrienols-rich fraction (TRF) was successfully entrapped in a self-assembled oil-in-water (O/W) emulsion system by using CaCO_3_ as the stabilizer. The emulsion droplet size, viscosity and tocotrienols entrapment efficiency (EE) were strongly affected by varying the processing (homogenization speed and time) and formulation (CaCO_3_ and TRF concentrations) parameters. Our findings indicated that the combination of 5000 rpm homogenization speed, 15 min homogenization time, 0.75% CaCO_3_ concentration and 2% TRF concentration resulted in a high EE of tocotrienols (92.59–99.16%) and small droplet size (18.83 ± 1.36 µm). The resulting emulsion system readily released the entrapped tocotrienols across the pH range tested (pH 1–9); with relatively the highest release observed at pH 3. The current study presents a potential pH-sensitive emulsion system for the entrapment and delivery of palm tocotrienols.

## 1. Introduction

Vitamin E is beneficial and important to health. It consists of two major groups of members, namely tocopherols and tocotrienols. Throughout the years, the extensive studies conducted on Vitamin E have only focused on tocopherols, particularly α-tocopherol, which is the most abundant form of Vitamin E derivative found in human and animal tissue [1]. However, tocotrienols have gained substantial prominence in recent times due to the discovery of their biological health effects. Aside from the lipophilic antioxidant activity, clinical results based on human studies suggest that tocotrienols can effectively lower blood cholesterol, suppress tumor growth, and have anti-inflammatory and anticancer effects [2]. Interestingly, tocotrienols are reported to possess better health impacts when compared with tocopherols. For instance, α-tocotrienol (but not α-tocopherol) was discovered to be responsible for the neuroprotective effect at a nanomolar level [1]. Considering the health-promoting benefits of tocotrienols, it is worth studying the possible application of tocotrienols in food for functional purposes. However, unlike tocopherols, tocotrienols are normally found at low concentration in most plant or food sources. Moreover, tocotrienols have very low bioavailability because they are naturally insoluble in water [3]. In addition, tocotrienols are reported to be more susceptible to degradation or oxidation than tocopherols [4]. Hence, it is necessary to come up with a way to protect these tocotrienols and improve their bioavailability.

One possible solution is the use of a Pickering emulsion system to encapsulate tocotrienols. Pickering emulsion is well known for its high stability; as a result of the stabilizing effect of solid particles on the oil-water interface of emulsion droplets. In this context, the solid particle acts as a stabilizer by forming a physical barrier that prevents the emulsion droplets from coalescing [5]. In recent years, there has been a growing interest in Pickering emulsion because it is generally considered as an environmental-friendly and biocompatible system in the sense that it can be formed without the use of synthetic surfactants. The utilization of natural, solid particles eliminates the risks of side effects, such as tissue irritation, commonly associated with the use of synthetic surfactants in conventional emulsion systems [6,7]. Numerous types of solid particles, for instance, hydrocolloids and proteins have been applied as the Pickering stabilizer [8]. Complexes resulted from the interaction between the colloidal particles may also be used to stabilize Pickering emulsions [9]. In a study done by Mathapa and Paunov [10], an O/W emulsion was successfully formed by utilizing the cyclodextrin-oil complexes. With the use of suitable types of stabilizing particles, Pickering emulsion can even be formed via low energy methods. This is demonstrated in a previous study carried out by our research group in which we successfully produced Pickering emulsions containing palm olein via different low energy homogenization methods (magnetic stirring, membrane and high-speed homogenization) by using CaCO_3_ particles as a stabilizer [11]. Thus, based on the success of our previous study, we believe that these self-assembly emulsification methods, particularly the high-speed homogenization method, can be employed to fabricate Pickering emulsion systems with the ability to entrap and deliver oil or bioactive compounds.

CaCO_3_ is readily available and commercially low in cost. There is a high demand for CaCO_3_ for various applications in the manufacturing of food, pharmaceutical and material products. Because of its biocompatibility, CaCO_3_ particle is safe for human consumption and harmless to the environment [12]. Moreover, CaCO_3_ is particularly responsive to changes in pH and readily soluble in acidic conditions. Due to these characteristics, CaCO_3_ has been used as a stabilizer in the preparation of emulsion systems with pH-triggered release functionality [13]. In some studies, CaCO_3_ is applied as a self-assembled stabilizer in the preparation of Pickering emulsions [14,15].

In the current study, we focused on the fabrication of an oil-in-water (O/W) Pickering emulsion system via a high-speed homogenization method to encapsulate palm tocotrienol rich fraction (TRF) by using CaCO_3_ as the stabilizer. Since the processing and formulation parameters are critical factors that determine the success of the formation of the Pickering emulsion, we have therefore identified and evaluated the effects of two processing parameters (homogenization speed and time) and two formulation parameters (the concentrations of CaCO_3_ and TRF) on the characteristics of the fabricated Pickering emulsions. Since CaCO_3_, a pH-sensitive material is used as the stabilizer, we were essentially fabricating a pH-responsive emulsion system that could release TRF in response to the changes in pH. For that reason, we further explored the release of tocotrienols from the resulting Pickering emulsions across a wide range of pH.

## 2. Materials and Methods

### 2.1. Materials

Palm TRF of approximately 50:50 tocotrienols-to-tocopherols ratio (133.77 mg/g α-tocotrienol, 180.94 β-/γ-tocotrienols, 72.23 mg/g δ-tocotrienol, and 249 mg/g α-tocopherol) was received from the Supervitamins Company (Johor, Malaysia). RBD palm olein (*Elaeis guineensis* var. *tenera*) containing a total of about 515.64 µg/g tocotrienols was obtained from Moi Foods Malaysia Sdn. Bhd. (Selangor, Malaysia). Precipitated CaCO_3_ was supplied by the NanoMaterials Technology Company (Singapore). The standard solution mixture consists of all eight isomers (α-, β-, γ-, δ-, tocotrienols and tocopherols) was purchased from LGC Standards (Teddington, UK).

### 2.2. Preparation and Characterization of CaCO_3_ Dispersion

The CaCO_3_ dispersion and subsequent Pickering emulsion were prepared by adopting the method described in a previous study [11]; wherein the precipitated CaCO_3_ was initially dispersed in deionized water using an Ultra Turrax rotor-stator homogenizer (IKA, Staufen, Germany) at 5000 rpm for 30 min to obtain an aqueous stock dispersion of 5% (*w*/*v*) CaCO_3_ concentration. The coarse dispersion was further processed using a high-pressure homogenizer (Microfluidizer M-110 L, Microfluidics, Westwood, MA, USA) at 22,000 psi for 4 passes. The final dispersion was diluted to the desired concentration using deionized water for further use in the Pickering emulsification process.

The morphology characteristic and the average size of CaCO_3_ particles were examined by using a high-resolution field emission scanning electron microscope (FESEM) coupled with a dispersive X-ray spectrometer (SU8010 model, Hitachi, Tokyo, Japan). The air-dried sample was coated with gold prior to analysis.

### 2.3. Homogenization Process

Palm TRF was dissolved into refined, bleached and deodorized (RBD) palm olein at a certain weight percentage. Then, the oil was stirred well and added (5%, *v*/*v*) into the diluted CaCO_3_ dispersion using a high-shear homogenizer (Silverson, MA, USA). The processing parameters were manipulated based on Table 1, whereby the homogenization speed was varied (2000, 3000, 4000 and 5000 rpm), followed by the homogenization time (5, 10, 15, 20, 25 min). The effect of CaCO_3_ was then studied by preparing different concentrations of CaCO_3_ solution (0.5, 0.625, 0.75, 0.875, 1.0%, *w*/*v*). Thereafter, the TRF concentration to be added into the oil phase was varied from 1 to 5% (*w*/*w*). The fabricated emulsion was allowed to settle for 30 min to observe for any occurrence of oiling off, creaming or sedimentation.

### 2.4. Emulsion Characterization

**Figure 1:**
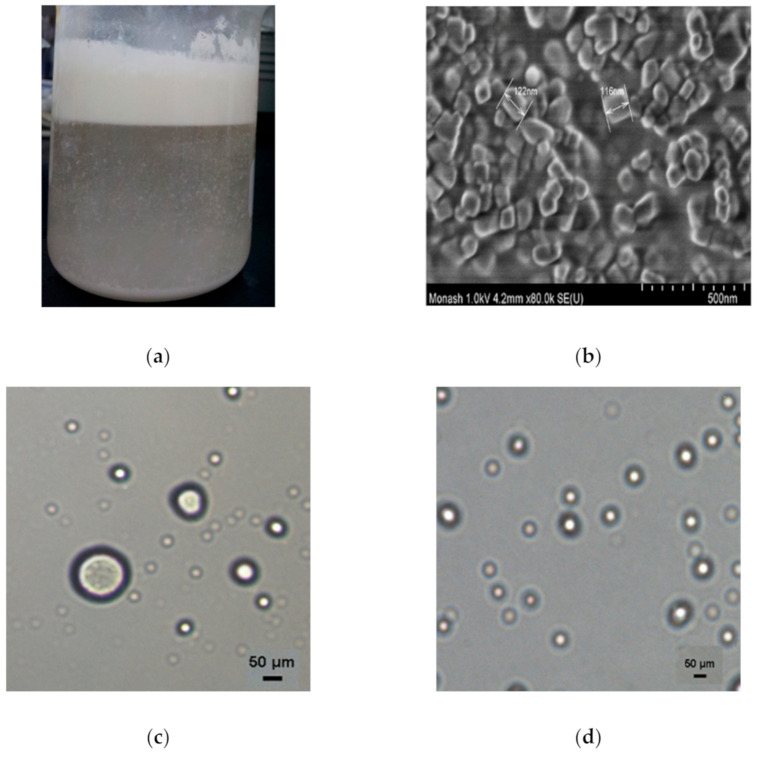

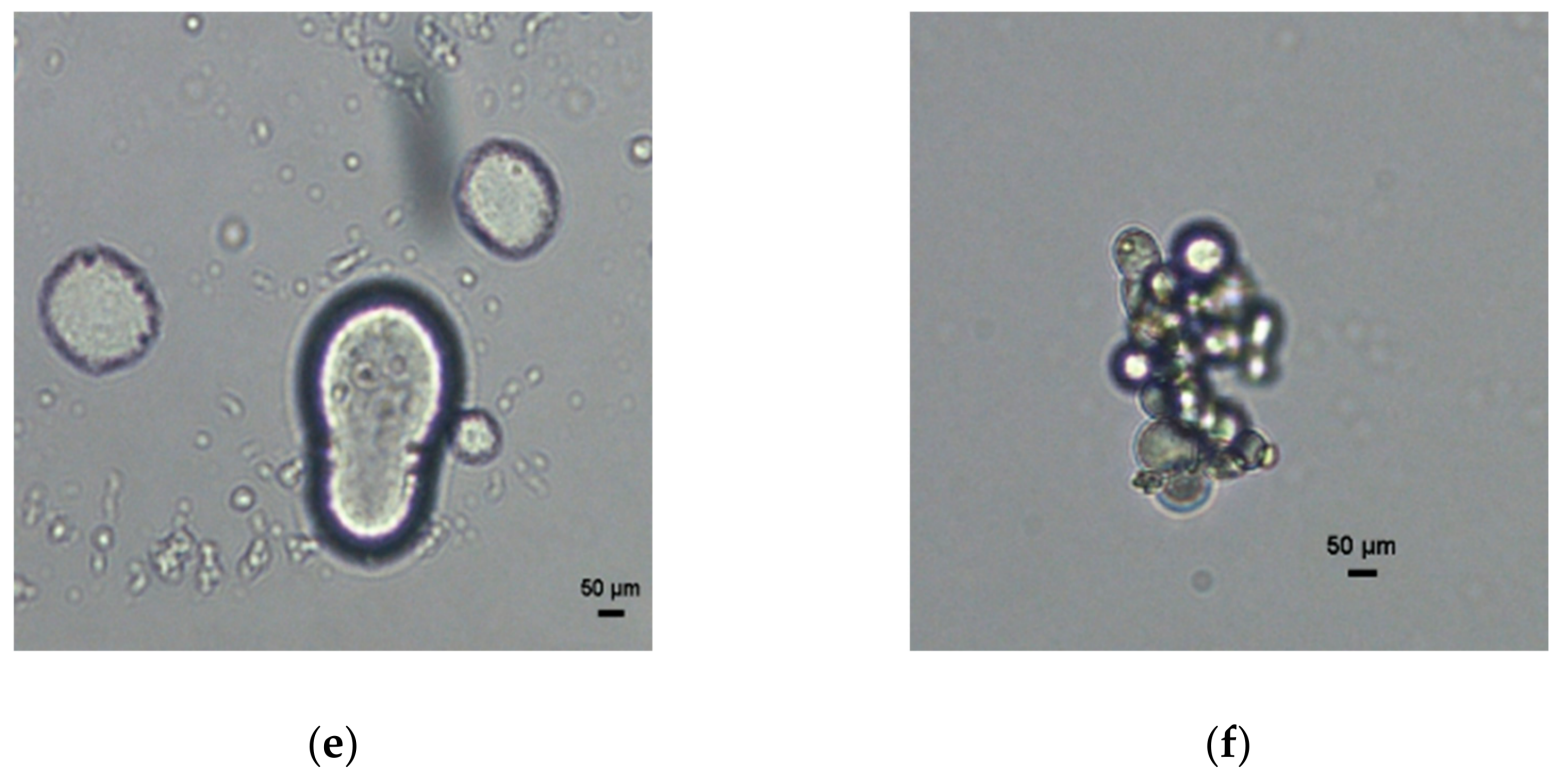
Images of (**a**) Freshly prepared tocotrienol rich fraction (TRF) emulsion (1% CaCO_3_); (**b**) field emission scanning electron microscope (FESEM) image of 1% CaCO_3_ dispersion; (**c**) microscopic image of initial TRF emulsion after storage (2000 rpm speed, 5 min time, 1% TRF content); (**d**) microscopic image of final obtained TRF emulsion after storage; (**e**) Coalescence of emulsion droplets resulted from 4000 rpm speed; (**f**) droplet aggregation of the sediment in TRF emulsion prepared by 1% CaCO_3_.

Basically, the resulting emulsion consists of two to three layers. The first layer on the top is the emulsified phase (EP), followed by the aqueous and sediment layers, if any (Figure 1a). As tocotrienols would be contained in the EP, EP was used for all of the analyses. The emulsion type was verified by using the drop test, whereby O/W emulsion would readily be dispersed in water; whilst the water-in-oil (W/O) emulsion would disperse in heptane. The droplet size distribution and mean diameter of the EPs were analyzed by laser light scattering using a Mastersizer 2000 equipped with a Hydro 2000 MU dispersion unit (Malvern Instruments Ltd., Worcestershire, UK). The analysis was carried out by dropping the diluted EP into the continuous phase of deionized water, which was agitated at 1500 rpm. The refractive indexes of deionized water and palm oil were set at 1.33 and 1.45, respectively. The volume-weighted mean diameter (d_4,3_) was used to represent the droplet mean diameter. The stability of the emulsion was evaluated by monitoring the changes in the droplet size and creaming index (CI) for 7 days at room temperature (25 °C). The CI was determined by the height of the EP against the total emulsion, as stipulated in the following Equation (1):

CI (%) = H_ep_/H_t_ × 100, (1)

where H_ep_ refers to the height of EP and H_t_ is the height of the total emulsion.

In the meantime, the stability of EP under centrifugal force was investigated by centrifuging approximately 4 g of the freshly prepared EPs at 2264× *g* for 15 min. The height of each phase (oil released, emulsion, aqueous and sediment) after centrifugation was measured and recorded.

The visual image of EP droplets was obtained by means of an optical light microscope (Nikon Eclipse 80i Binocular, Melville, NY, USA). The EP was diluted ten times using deionized water and dropped onto a glass slide covered with a coverslip to be viewed at 40× magnification. The image was captured using a CCD camera (Nikon 5 megapixel, Kanagawa, Japan).

### 2.5. Emulsion Viscosity

The viscosity of the EP was analyzed using a rheometer equipped with a cylinder measuring system (Rheolab QC, Anton Paar, Ashland, VA, USA). The measurement was performed at a constant shear rate of 1559 s^−1^ and a temperature of 25 °C, with the data reported in mPa.s.

### 2.6. Tocotrienol Determination

The final emulsion (2 g) was added into 10 mL sodium phosphate solution and the pH adjusted using 1 M HCl and 1 M NaOH to the desired final value (pH 1, 3, 5, 7, 9) to study the trigger effect of different pHs on tocotrienols release. After 1 h, 1 mL of the sodium phosphate solution was taken to analyze the number of tocotrienols released.

#### 2.6.1. Extraction

The determination of tocotrienols content in the oil and emulsion samples was performed according to Xu [16] and Xu, Harvey, Pavlina, Zaloga, and Siddiqui [17], with slight modification. In brief, 0.1 g of TRF or palm olein was extracted with 10 mL hexane and vortexed. The hexane layer was purged to dryness using nitrogen gas and the dried residue was dissolved in acetonitrile and filtered (0.45 µm nylon syringe filter) prior to HPLC analysis.

As for emulsion or buffer medium samples, 0.2 g of the sample was added with 2 mL hexane (0.05% 2, 6-Di-tert-butyl-4-methylphenol) and 800 µL of methanol. The mixture was vortexed for 1 min and centrifuged at 1400× g for 20 min. The hexane layer was collected and the extraction procedure was repeated again. The hexane layers were combined and evaporated to dryness by nitrogen gas. The extract was reconstituted using acetonitrile and filtered with a nylon syringe filter (0.45 µm) before being subjected to HPLC analysis.

#### 2.6.2. HPLC Analysis

A Shimadzu HPLC system equipped with a fluorescence detector (Prominence LC-20AD, Shimadzu, Kyoto, Japan) was used for the detection of tocotrienols. The separation was carried out with an isocratic flow of methanol/acetonitrile/dichloromethane (25:23:2, *v*/*v*/*v*) at a flow rate of 1 mL/min, and eluted through a reverse phase C18 column (250 × 4.6 mm, 5 µm, Phenomenex, Torrance, CA, USA). The temperature of the column was maintained at 30 °C and the injection volume was 25 µL. The excitation and emission wavelengths for the fluorescence detector were set at 290 and 330 nm, respectively.

Quantification of the three isomers of tocotrienols (α-, β-/γ-, and δ-) was done by referring to each of the external calibration curves (R^2^ ≥ 0.99) established for the individual isomer using the reference standard mixture. The standard solutions of 2 to 10 µg/mL were prepared through serial dilution of the standard mixture with acetonitrile. Tocotrienols content was calculated from the triplicate injections by using the linear calibration curves. The final content was expressed in µg/g sample. The entrapment efficiency of tocotrienols was calculated based on Equation (2) as follow:

Entrapment efficiency of tocotrienols (%) = T_sample_/T_oil_ × 100, (2)

where T_sample_ refers to the tocotrienols content in the sample, whilst T_oil_ is the total tocotrienols amount in the oil (palm olein and TRF) added into the emulsion.

### 2.7. Statistical Analyses

The entire experimental measurements of the analyses were conducted in triplicates on duplicate samples. The data were expressed as mean ± standard deviation, and the significant difference among the data was determined at *p* < 0.05 by one-way analysis of variance (ANOVA) using the Minitab Software (released 16.2.2; Minitab Inc., State College, PA, USA).

## 3. Results and Discussion

As depicted in Figure 1b, the CaCO_3_ dispersion containing particles with a homogenous shape and size of approximately 100 nm was successfully prepared through high-pressure homogenization. Because of their hydrophilic nature, the adsorption of these submicron-sized particles on the oil droplets contributed to the formation of O/W emulsions [18]. The droplets containing emulsified TRF formed the cream emulsified layer on the top (Figure 1a), providing the creaming characteristic normally observed in Pickering emulsions [19].

### 3.1. Effect of Homogenization Speed

Based on our preliminary work (result not shown), when the homogenization speed was below 2000 rpm, EP failed to form as the speed was not sufficient to homogenize the oil and CaCO_3_ dispersion. On the other hand, homogenization speed above 5000 rpm converted the O/W emulsion to a W/O emulsion. As such, the range of homogenization speed used in this study was fixed at 2000 to 5000 rpm. This range of homogenization speed was found to produce O/W emulsions with the monodisperse distribution. Our findings showed that an increase in homogenization speed from 2000 to 5000 rpm improved the emulsion homogeneity (span value reduced from 0.59 ± 0.03 to 0.40 ± 0.07) and significantly (*p* < 0.05) reduced the droplet size by half (Table 2).

**Figure 2:**
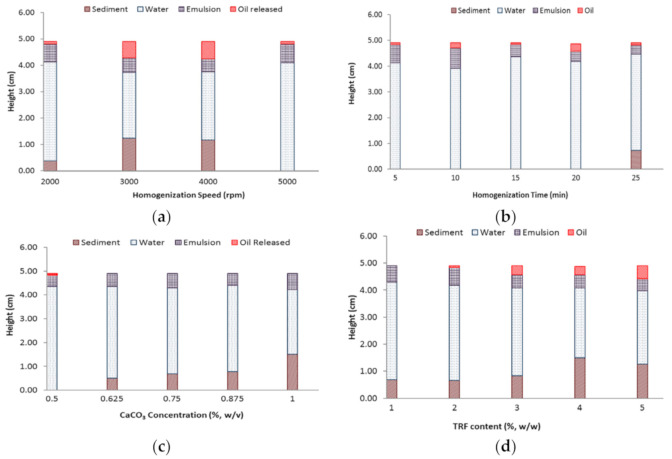
Emulsified phase (EP) height (cm) after centrifugation as affected by (**a**) homogenization speed (rpm); (**b**) homogenization time (min); (**c**) CaCO_3_ concentration (%, *w*/*v*); (**d**) TRF content (%, *w*/*w*).

During the emulsification process, particle adsorption/desorption and droplet breakup/coalescence simultaneously occur until equilibrium is achieved [20]. By increasing the homogenization speed, the number of smaller droplets increased. The CaCO_3_ particles must then be fast enough to adsorb onto these droplets’ surface to achieve optimum droplet coverage to suppress droplet coalescence [21]. The increase in energy facilitates the particle adsorption rate by promoting the collision of the particles and droplets [22], which therefore reduced the droplet size efficiently. This speed increment, however, did not result in a consistent trend on both the droplet size and creaming stability. In fact, the droplet size of the emulsion formed at 4000 rpm was rather high. Lower creaming stability of EPs was observed when homogenization speeds of 2000 to 4000 rpm were applied, particularly at 4000 rpm, as shown by its lower CI and higher extent of emulsion break down (Figure 2a). In contrast, a more stable Pickering emulsion was produced at 5000 rpm, with a smaller increase in the mean droplet size (approximately 35%) observed over a one-week period (Table 2).

**Figure 3:**
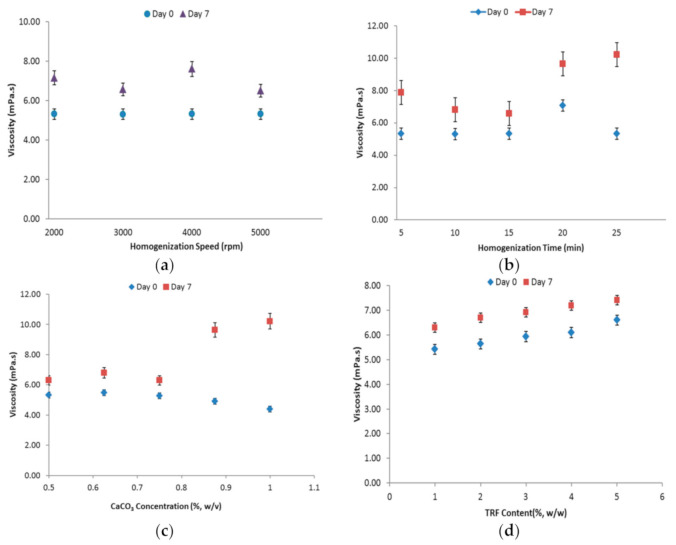
Viscosity of EPs prepared using different (**a**) homogenization speed (rpm); (**b**) homogenization time (min); (**c**) CaCO_3_ concentration (%, *w*/*v*); (**d**) TRF content (%, *w*/*w*).

The change in homogenization speed did not impose any notable change in the viscosity of the EPs (Figure 3a). However, we discovered a significant (*p* < 0.05) increase in viscosity after the one week-storage period, with the highest increase observed for the emulsion homogenized at 4000 rpm. We believe that the increase may have been caused by the lower emulsion stability. In addition, the interaction between the CaCO_3_ particles may also contribute to the viscosity increase during storage, as Ca^2+^ ions tend to promote droplet flocculation and cause an increase in the strength of droplet-droplet attraction [23]. In consideration of the smaller droplet size and higher stability of the emulsion obtained, a homogenization speed of 5000 rpm was selected as the most suitable homogenization speed for the fabrication of tocotrienols Pickering emulsion.

### 3.2. Effect of Homogenization Time

By applying a speed of 5000 rpm, the increase in the process time from 5 to 25 min broadened the size distribution with approximately three time increments in span value from 0.40 ± 0.07 to 1.29 ± 0.11. However, according to the result shown in Table 2, the time of 15 min at the speed of 5000 rpm led to a smaller size range with a three-fold reduction in droplet size. Further process of 20 min duration gave rise to a larger mean droplet size, which then dropped again at 25 min, thus implying destabilization of the emulsion system. In contrast with the other levels (10, 20 and 25 min), the EP produced at 15 min experienced a slight change in droplet size and CI throughout the one-week storage. Apparently, the emulsions formed by using 10, 20 and 25 min homogenization time resulted in lower stability against coalescence, as supported by the lower CI and emulsion breakup upon centrifugation (Table 2 and Figure 2b). In view of the adsorption kinetics, we could deduce that the homogenization time of 15 min was sufficient for the particles to achieve stable adsorption, hence forming a more stable emulsion with higher entrapment efficiency (Table 3) [24]. Homogenization time beyond 15 min, on the other hand, may cause destabilization of the EP.

The significant (*p* < 0.05) increase in the viscosity of freshly-prepared EP at 20 min (Figure 3b) may be explained by the lower emulsion stability. This lowered stability was probably one of the main reasons accounting for the higher viscosity change in the emulsions prepared using homogenization times of 20 and 25 min, after storage. On top of that, the droplet aggregation resulting from the inter-particle interaction may also contribute to the increase in emulsion viscosity. According to the findings, the emulsion resulted from a 15 min homogenization duration showed lesser viscosity change among all. Thus, a homogenization time of 15 min was chosen as the optimized time to be used in the subsequent phases of this study.

### 3.3. Effect of CaCO_3_ Concentration

In line with previous studies [19,25], the volume fraction of the EP increased with elevated CaCO_3_ level. As expected, an elevation of the CaCO_3_ particle concentration from 0.5 to 1% (*w*/*v*) contributed to an approximately five-fold reduction in the mean droplet size (Table 2). According to the limited coalescence phenomenon theory by Arditty, Whitby, Binks, Schmitt, and Leal-Calderon [26], the droplets tend to coalesce until the coverage limit of the droplets is achieved, provided that a limited number of particles are available in the emulsion system. In other words, a higher content of CaCO_3_ could supply more particles for droplet adsorption, thereby facilitating the full coverage of more tiny droplets. Because of the enhanced coverage of the emulsion droplets, the resulting EPs exhibited improved stability with improved tocotrienols entrapment efficiency (Table 3). In particular, the emulsions of 0.875% and 1% were found to be exceptionally stable with higher CI and the smallest change in droplet size (Table 2). The emulsions of higher CaCO_3_ content were discovered to demonstrate higher resistance against centrifugation (Figure 2c). However, the elevated CaCO_3_ level beyond 0.75% (0.875% and 1%) resulted in the sedimentation of the emulsion (Figure 1a), which was most likely caused by an excess of CaCO_3_ and/or the higher density of droplets. The density of the droplets will increase with higher particle concentration and eventually sink when the droplets become denser than the continuous aqueous phase [27]. In addition, a further increase in the CaCO_3_ level may also result in droplet aggregation (Figure 1f) and therefore cause sedimentation of the droplets.

For freshly prepared emulsion (Day 0), the increase of the CaCO_3_ level above 0.75% slightly reduced the emulsion viscosity (Figure 3c). This observation was most likely attributable to the droplet size reduction. However, the viscosity was revealed to increase after one week of storage, in which the increment became larger for emulsions with increased CaCO_3_ content beyond 0.75%. With more particles available in the system, the higher particle-particle interaction was again suspected to account for the higher viscosity after storage. Despite the higher stability of 0.875 and 1% CaCO_3_ EPs, the two levels imposed droplet aggregation and sedimentation in the emulsions. Thus, CaCO_3_ concentration of 0.75% that contributed to small droplet size and acceptably high emulsion stability was chosen to be applied in subsequent experiments.

### 3.4. Effect of TRF Concentration

The increase in TRF concentration in the oil phase from 1 to 4% caused a progressive increment of droplet size. A phase inversion of the emulsion from O/W to W/O was observed as the TRF concentration increased to 5%. Generally, phase inversion of Pickering emulsion can occur due to changes in the composition of the dispersed and continuous phases [28]. Similar to the effect of homogenization speed, this change of phase-type was again caused by the irreversible interaction between the particles on the droplets. Additionally, an increase in TRF concentration resulted in an increase in the viscosity of the oil phase, thereby lowering the particle adsorption rate. In this scenario, the initial particle adsorption force would be weaker, thus inducing particle detachment and consequently, droplet coalescence [29]. Given that the particle adsorption kinetics was affected, the emulsion stability was reduced with increasing oil viscosity, as indicated by the droplet size increase and the reduction in CI after 7 days (Table 2). Moreover, higher amounts of oil and sediment were formed with increasing TRF levels due to emulsion breakdown under centrifugation (Figure 2d). As a result, the tocotrienols entrapment efficiency was negatively affected (Table 3). Based on Figure 3d, the reduced emulsion stability may have caused the progressive increase in the emulsion viscosity. Aside from the stability factor, the increased particle interaction and droplet aggregation may have also contributed to the viscosity increase after storage. Taking everything into consideration, an emulsion containing 2% TRF appeared to be acceptably stable and suitable to be applied as the ideal level for further preparations of TRF emulsion.

### 3.5. Effect of pH on Tocotrienol Release

Theoretically, the release of the interior oil content is triggered by acidic conditions, as the adsorbed CaCO_3_ particles readily dissolve at reduced external pH [13]. The result summarized in Table 4 proved the pH-dependent release of tocotrienols of the TRF emulsion. In particular, the dissolution rate of CaCO_3_ was relatively the highest at pH 3 as supported by the largest amount of tocotrienols being released within the fixed period of time. Although the release of tocotrienols was shown to be the highest (relatively) at pH 3, one should take note that the actual total release at this pH was only 3.73%. However, we believe that this low percentage of release was due to the sensitive nature of the tocotrienols. As described in Section 2.5, we adjusted the pH of the emulsion to a desired final value and allowed the release of tocotrienols to occur for 1 h. Then, after 1 h, the tocotrienols concentration was determined. During this 1 h period, we suppose that the released tocotrienols degraded, thus only a small amount of tocotrienols remained to be detected. This, in turn, translates to a very low percentage of tocotrienol release. An even lower amount of tocotrienols was present in the medium as the external pH was further reduced to pH 1. At this extremely acidic condition, the emulsion system totally broke down and the tocotrienols underwent rapid degradation, thereby contributing to the low presence of tocotrienols found in the external medium. The progressive increment from pH 3 to 7 slowed down the release rate of tocotrienols, as revealed by the significantly (*p* < 0.05) lower amount of tocotrienols released from the emulsion. Interestingly, the number of tocotrienols released at pH 9 was relatively higher than those at pH 3 to 7. However, we believe that this is due to the high amount of OH^−^ present in the medium, which caused the dissociation of CaCO_3_ to calcium hydroxide. Subsequently, desorption of the particles from the oil droplets occurred, leading to the release of tocotrienols. Overall, the final TRF emulsion not only exhibited responsiveness towards pH change, but also presented a high tocotrienols entrapment efficiency (81.46 ± 1.91%). Unlike the reported methods which require modification or reaction of solid particles for better stabilizing effect [9,10], the present emulsification method that utilizes unmodified CaCO_3_ nanoparticles is able to contribute to a satisfying entrapment efficiency without additional processing of the particles. Furthermore, in contrast to the other high-energy emulsification methods, such as the high-pressure homogenization [30], the emulsification method that we applied is more energy-saving and thus, economic.

## 4. Conclusions

The present findings revealed the suitability of CaCO_3_ particles as a pH-sensitive stabilizer in forming an edible food Pickering emulsion for palm TRF. Ideally, the increase in the energy input (in terms of the homogenization speed), along with a moderate homogenization time, would be preferable to improve the quality of emulsion. Under controlled processing conditions, the droplet size reduction and particularly the stability of EP would then be governed by the particle concentration, whereby a higher CaCO_3_ concentration, up to a certain extent, would further reduce the droplet size and increase the stability of the emulsion. The higher CaCO_3_ particle content (0.75%) was revealed to be able to stabilize and entrap a higher content of TRF (2%). Owing to the pH-sensitivity of CaCO_3_ particles, the reduction of the environmental pH would increase the dissolution rate of CaCO_3_ and therefore speed up the release of the tocotrienols within a fixed amount of time. Considering the possible decomposition of tocotrienols, the EP should not be applied in extremely acidic conditions (pH 1). In view of the acceptable emulsion quality, the outcome of this study indicated that the resulting emulsion could serve as a TRF encapsulation and/or delivery system for future applications in the food and nutraceutical industries.

## Figures and Tables

**Table 1:** Processing parameters of tocotrienol rich fraction (TRF) emulsions.

Stage	Homogenization Speed (rpm)	Time (min)	CaCO_3_ Concentration (%, *w*/*v*)	Tocotrienol Content (%, *w*/*v*)
I	2000	5	0.5	1
	3000			
	4000			
	5000			
II	Optimized speed	5	0.5	1
		10		
		15		
		20		
		25		
III	Optimized speed	Optimized time	0.5	1
			0.625	
			0.75	
			0.875	
			1.0	
IV	Optimized speed	Optimized time	Optimized CaCO_3_ concentration	1
				2
				3
				4

**Table 2:** Droplet mean size and creaming index (CI) of freshly prepared (Day 0) and stored emulsified phases *.

Parameters	Day 0		Day 3		Day 7	
	Mean Size (μm)	CI	Mean Size (μm)	CI	Mean Size (μm)	CI
Homogenization Speed (rpm)						
2000	63.69 ± 0.85 ^a,B^	38.67 ± 2.07 ^b,A^	68.30 ± 1.80 ^a,B^	30.67 ± 2.07 ^a,b,B^	79.69 ± 0.85 ^b,A^	30.67 ± 2.07 ^a,b,B^
3000	40.78 ± 0.81 ^c,C^	40.67 ± 1.63 ^b,A^	47.59 ± 1.92 ^b,B^	26.00 ± 3.35 ^c,B^	73.05 ± 6.88 ^b,A^	26.00 ± 3.35 ^c,B^
4000	50.06 ± 1.21 ^b,C^	47.33 ± 1.63 ^a,A^	82.38 ± 5.11 ^a,B^	27.33 ± 1.63 ^b,c,B^	113.34 ± 10.09 ^a,A^	27.33 ± 1.63 ^b,c,B^
5000	33.00 ± 0.56 ^d,C^	40.67 ± 1.63 ^b,A^	35.72 ± 0.24 ^c,B^	32.67 ± 1.63 ^a,B^	44.55 ± 1.87 ^c,A^	32.67 ± 1.63 ^a,B^
Homogenization Time (min)						
5	33.00 ± 0.56 ^a,C^	40.67 ± 1.63 ^a,A^	35.72 ± 0.24 ^a,B^	32.67 ± 1.63 ^a,B^	44.55 ± 1.87 ^a,A^	32.67 ± 1.63 ^a,B^
10	28.38 ± 1.42 ^b,C^	38.67 ± 2.07 ^a,A^	34.19 ± 0.83 ^b,B^	26.67 ± 2.07 ^b,B^	42.56 ± 3.73 ^a,A^	26.67 ± 2.07 ^b,B^
15	24.56 ± 0.35 ^c,C^	38.67 ± 2.07 ^a,A^	28.62 ± 0.41 ^c,B^	30.40 ± 2.53 ^a,B^	34.81 ± 2.38 ^b,A^	30.40 ± 2.53 ^a,B^
20	27.99 ± 0.24 ^b,C^	38.67 ± 2.07 ^a,A^	32.92 ± 0.98 ^b,B^	18.93 ± 1.73 ^c,B^	43.26 ± 5.49 ^a,A^	18.93 ± 1.73 ^c,B^
25	23.36 ± 1.67 ^c,C^	39.33 ± 1.63 ^a,A^	28.18 ± 0.45 ^c,B^	30.67 ± 2.07 ^a,B^	36.39 ± 2.38 ^b,A^	30.67 ± 2.07 ^a,B^
CaCO_3_ Concentration (%, w/v)						
0.5	24.56 ± 0.35 ^a,C^	38.67 ± 2.07 ^c,A^	26.41 ± 0.90 ^a,B^	30.40 ± 2.53 ^c,B^	34.81 ± 2.38 ^b,A^	30.40 ± 2.53 ^c,B^
0.625	22.43 ± 1.48 ^b,B^	47.78 ± 2.45 ^b,A^	25.29 ± 0.88 ^b,B^	39.44 ± 2.45 ^b,B^	40.46 ± 5.67 ^a,A^	39.44 ± 2.45 ^b,B^
0.75	18.65 ± 0.83 ^c,B^	48.61 ± 2.02 ^b,A^	18.87 ± 0.41 ^c,B^	40.69 ± 2.07 ^b,B^	20.58 ± 1.08 ^c,A^	40.69 ± 2.07 ^b,B^
0.875	15.09 ± 0.84 ^d,B^	50.97 ± 2.76 ^a,b,A^	15.94 ± 0.63 ^d,B^	46.53 ± 2.81 ^a,B^	18.06 ± 1.84 ^c,A^	46.53 ± 2.81 ^a,B^
1.0	11.58 ± 0.64 ^e,B^	53.89 ± 2.67 ^a,A^	13.00 ± 0.41 ^e,B^	49.44 ± 2.77 ^a,B^	15.76 ± 0.99 ^c,A^	49.44 ± 2.77 ^a,B^
Tocotrienol Content (%, w/w)						
1	18.65 ± 0.83 ^c,B^	48.61 ± 2.02 ^a,A^	18.43 ± 0.51 ^d,B^	40.69 ± 2.07 ^a,B^	20.58 ± 1.08 ^c,A^	40.69 ± 2.07 ^a,B^
2	18.83 ± 1.36 ^c,B^	47.22 ± 3.56 ^a,A^	19.56 ± 0.87 ^c,B^	38.75 ± 3.28 ^a,B^	24.07 ± 1.44 ^b,A^	38.75 ± 3.28 ^a,b,B^
3	20.61 ± 0.18 ^b,B^	46.53 ± 2.86 ^a,A^	20.73 ± 0.20 ^b,B^	38.33 ± 3.12 ^a,B^	26.02 ± 3.52 ^b,A^	38.33 ± 3.12 ^a,b,B^
4	24.92 ± 0.92 ^a,B^	46.67 ± 2.93 ^a,A^	25.44 ± 0.38 ^a,B^	37.78 ± 3.36 ^a,B^	31.34 ± 1.46 ^a,A^	33.75 ± 3.86 ^b,B^

* The reported values represent mean ± standard deviation (*n* = 6). ^a–e^ Different lowercase in the same column are significant different (*p* < 0.05). ^A–C^ Within the same row for comparison among days, different uppercases are significant different (*p* < 0.05).

**Table 3:** Tocotrienol entrapment efficiency *.

Parameters				Entrapment Efficiency (%)			
Speed (rpm)	Time (min)	CaCO_3_ Content (%, *w*/*v*)	TRF Content (%, *w*/*w*)	α-Tocotrienol	β-/γ-Tocotrienols	δ-Tocotrienol	Total Tocotrienols
2000	5	0.50	1	34.15 ± 0.61 ^d^	37.44 ± 1.97 ^d^	50.01 ± 1.71 ^d^	38.44 ± 0.76 ^d^
5000	5	0.50	1	39.88 ± 0.44 ^c^	51.50 ± 0.81 ^c^	68.93 ± 0.97 ^c^	50.72 ± 0.67 ^c^
5000	15	0.50	1	83.09 ± 4.65 ^b^	78.12 ± 2.92 ^b^	80.82 ± 8.66 ^b^	80.37 ± 3.79 ^b^
5000	15	0.75	1	99.16 ± 1.14 ^a^	93.88 ± 2.59 ^a^	92.59 ± 4.21 ^a^	95.49 ± 1.42 ^a^
5000	15	0.75	2	87.55 ± 3.49 ^b^	75.17 ± 1.83 ^b^	86.18 ± 1.83 ^a,b^	81.46 ± 1.91 ^b^

* The reported values represent mean ± standard deviation (*n* = 6). ^a–d^ Different lowercase in the same column are significant different (*p* < 0.05).

**Table 4:** Release of tocotrienols in different pH medium *.

pH	α-Tocotrienol		β-/γ-Tocotrienols		δ-Tocotrienol		Total Tocotrienols	
	Concentration (µg/g)	% Release	Concentration (µg/g)	% Release	Concentration (µg/g)	% Release	Concentration (µg/g)	% Release
1	N/A	-	2.42 ± 0.06 ^c^	0.37 ± 0.01 ^c^	1.88 ± 0.06 ^d^	0.62 ± 0.02 ^d^	4.30 ± 0.10 ^c^	0.29 ± 0.01 ^c^
3	4.83 ± 0.29 ^a^	0.89 ± 0.03 ^a^	8.86 ± 0.26 ^a^	1.35 ± 0.04 ^a^	4.52 ± 0.20 ^a^	1.49 ± 0.07 ^a^	18.25 ± 0.61 ^a^	1.21 ± 0.04 ^a^
5	N/A	-	1.76 ± 0.07 ^e^	0.27 ± 0.01 ^e^	2.27 ± 0.09 ^c^	0.75 ± 0.03 ^c^	4.04 ± 0.13 ^c^	0.27 ± 0.01 ^c^
7	N/A	-	2.10 ± 0.11 ^d^	0.32 ± 0.02 ^d^	1.89 ± 0.10 ^d^	0.62 ± 0.03 ^d^	3.98 ± 0.12 ^c^	0.26 ± 0.01 ^c^
9	2.94 ± 1.69 ^b^	0.54 ± 0.03 ^b^	6.45 ± 0.24 ^b^	0.98 ± 0.04 ^b^	4.10 ± 3.30 ^b^	1.35 ± 0.04 ^b^	13.51 ± 0.49 ^b^	0.90 ± 0.03 ^b^

* The reported values represent the mean ± standard deviation of six measurements from two replications. ^a–e^ Different lowercase in the same column are significant different (*p* < 0.05). N/A Not available.
